# Effects of salinity on the transcriptome of growing maize leaf cells point at cell-age specificity in the involvement of the antioxidative response in cell growth restriction

**DOI:** 10.1186/1471-2164-14-24

**Published:** 2013-01-16

**Authors:** Michael Kravchik, Nirit Bernstein

**Affiliations:** 1 Institute of Soil Water and Environmental Sciences, Volcani Center, POB 6, 50-250, Bet-Dagan, Israel

**Keywords:** Antioxidative response, Growth, Leaf, Maize, ROS, Salinity, Salt Stress

## Abstract

**Background:**

Salinity inhibits growth and development of most plants. The response to salinity is complex and varies between plant organs and stages of development. It involves challenges of ion toxicities and deficiencies as well as osmotic and oxidative stresses. The range of functions affected by the stress is reflected in elaborate changes to the transcriptome. The mechanisms involved in the developmental-stage specificity of the inhibitory responses are not fully understood. The present study took advantage of the well characterized developmental progression that exists along the maize leaf, for identification of salinity induced, developmentally-associated changes to the transcriptome. Differential subtraction screening was conducted for cells of two developmental stages: from the center of the growth zone where the expansion rate is highest, and from older cells at a more distal location of the growing zone where the expansion rate is lower and the salinity restrictive effects are more pronounced. Real-Time PCR analysis was used for validation of the expression of selected genes.

**Results:**

The salinity-induced changes demonstrated an age-related response of the growing tissue, with elevation of salinity-damages with increased age. Growth reduction, similar to the elevation of percentage dry matter (%DM), and Na and Cl concentrations were more pronounced in the older cells. The differential subtraction screening identified genes encoding to proteins involved in antioxidant defense, electron transfer and energy, structural proteins, transcription factors and photosynthesis proteins. Of special interest is the higher induced expression of genes involved in antioxidant protection in the young compared to older cells, which was accompanied by suppressed levels of reactive oxygen species (H_2_O_2_ and O_2_^-^). This was coupled with heightened expression in the older cells of genes that enhance cell-wall rigidity, which points at reduced potential for cell expansion.

**Conclusions:**

The results demonstrate a cell-age specificity in the salinity response of growing cells, and point at involvement of the antioxidative response in cell growth restriction. Processes involved in reactive oxygen species (ROS) scavenging are more pronounced in the young cells, while the higher growth sensitivity of older cells is suggested to involve effects on cell-wall rigidity and lower protein protection.

## Background

Salinity reduces growth and development of most plant species. Ion toxicity, deficiency, ion imbalance, as well as osmotic and oxidative stresses accompany salt stress and cause plant growth restriction [1-3]. Salinity affects the plant at all levels of organization: organ, tissue and cell [4,5]. The response to salinity is thereby complex, involving specificity at the organ and cell levels and variability with developmental stage and age [2,6,7]. Very little information is available concerning the mechanisms and factors involved in the interaction between the response to salinity and the developmental stage of the plant tissue, and the mechanisms involved in restriction of leaf growth and shoot development are not yet fully understood [8]. Identification of changes involved in processes of growth and development can be aided by spatial and temporal studies, focusing on growing organs, tissues, and cells at defined stages of development [7,9,10].

The array of functions which are affected by salt stress on the whole plant and the cellular levels are reflected by a complexity of changes in the transcriptome and the proteome [6,11]. Differential subtraction screening of Arabidopsis seedlings allowed identification of 84 salt-regulated genes, and characterization of the SOS signaling pathway that mediates ion homeostasis and contributes to salt tolerance [12]. In maize roots 11% of the genes were affected by salinity and most of the affected genes were related to transport and signal transduction pathways [13]. Differential subtraction screening and microarray analysis identified differences in the initial responses of salt-tolerant and salt-sensitive tomato cultivars and allowed isolation of transcription factors and genes involved in SOS pathway that were differently affected by salinity and consequently can affect plant salt tolerance [14]. Additionally, Qing et al. [15] identified differences in NaCl effect on the transcriptome of leaves and roots at the initial phase of the stress, which demonstrated that leaves were affected by the osmotic component of the stress, while roots were influenced by water stress and Na^+^ accumulation. Thus, salinity affects gene expression differently at various stages of tissue development and plant organs [6,13,15].

Recently, the involvement of reactive oxygen species (ROS) in the growth response of leaves to NaCl is gaining interest [7,9,16]. Salinity-stimulated increase in ROS may induce localized tissue damage [16], while reduced ROS concentrations in growing cells under salinity was suggested to restrict leaf elongation due to effects on cell-wall loosening [7,9]. Differences between the oxidative response of roots and shoots, as well as growing and mature leaf cells to salinity were identified [7] and indicated differential roles for various ROS scavenging enzymes at different cell developmental stages. Furthermore, the ameliorative effect of supplemental calcium on growth under salinity was suggested to take effect through modulating the antioxidative response as well as ROS levels [17].

The maize leaf is a good system for the study of stress effects on growth processes. Similar to most grass leaves, cell production and expansion in this leaf are restricted to a confined region at the leaf base, i.e., the growth zone [4,18]. This zone is characterized by a well-defined spatial gradient of cell development [4] along which salt effects are not identical, but demonstrate a characteristic response curve with cell developmental progression [4,19]. This system facilitates experimental sampling of tissue of unified developmental stage in the control and the salt treatments, thus preventing complications from interpretation of experimental results from tissues that differ in developmental stage [4]. The objective of the present study was to take advantage of the cell developmental progression along the leaf growing zone, to explore changes in gene expression under salinity, for identification of development-associated growth damage and tolerance mechanisms in the maize leaf. Differential subtraction screening was used to study salinity effects on the transcriptome at two developmental stages of growing leaf cells. The cDNA subtraction libraries were constructed for cells from the center of the growing tissue where the expansion rate is highest, and for older cells at a more distal portion of the growing zone, where the expansion rate is lower and the salinity restrictive effects are more pronounced. Comparison of salinity effects on the transcriptome at these two stages of cell development supported a role for processes involved in ROS scavenging and cell-wall rigidity determination in the extent of salinity response of the growing cells.

## Results

### Effect of salinity on spatial distribution of growth along the leaf

**Figure 1:**
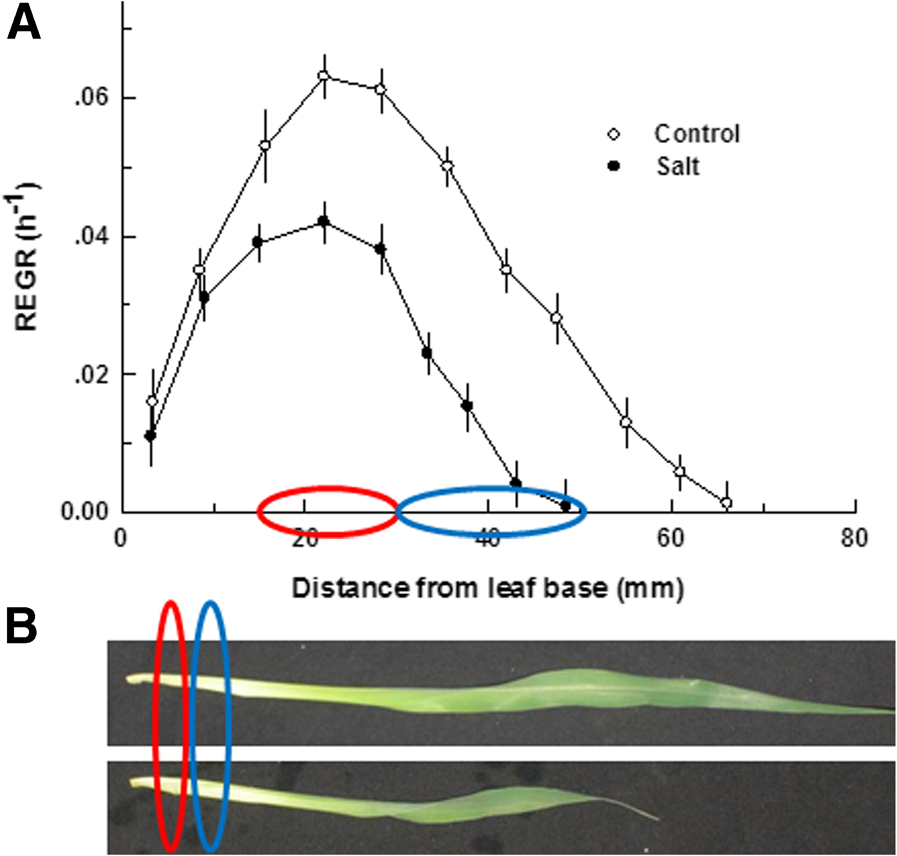
**Effect of salinity on maize leaf growth. A**. spatial distribution of REGR along the elongation zone of the leaf. The colored ovals on the x axis represent the two sections of the growing tissue sampled for the SSH analysis. Data were evaluated from prick hole marking experiments of leaf 4 of maize. If not shown, error bars are smaller than the symbol size. **B**. A photo of leaf 4 of maize from control (top-image) and salt-stressed plants (bottom-image) at the time of sampling for the physiological analysis and SSH analysis. The plants were grown at 1 mM NaCl (control) or 80 mM NaCl (salt).

Salinity exerted a characteristic effect on plant development and leaf growth [4]. Shoot biomass was reduced by 43.1% under salinity and the rate of leaf elongation was reduced by 49.3% (Tables 1 and 2). The elongation zone, that is located at the leaf base, was shortened under salinity. The intensity of elongation throughout the central and distal portions of the elongation zone were reduced (Figure 1). The tissues located at the center of the growing zone, 15–30 mm from the leaf base- which include the region of highest growth intensity, and the older growing tissue located 30–50 mm from the leaf base- which demonstrates reduced growth, were selected for SSH analyses. In both locations, salinity reduced elongation- by 33.9 and 66.7% in the center and distal parts of the elongation zone, respectively. The reduction was significantly higher for the distal part of the elongation zone (P<0.05). In accord with the higher restrictive affects of salinity on elongation of older compared to younger cells, the salinity-induced elevation of % DM, and contents of Na were also more pronounced in the older cells. % DM was elevated by 38.8% and 48.6% in the locations 15–30 mm and 30–50 mm from leaf base, respectively; Na content increased by 42.0 μmol g FW^-1^ and 52.9 μmol g FW^-1^ in the locations 15–30 mm and 30–50 mm from leaf base, respectively (Tables 1 and 2). The elevation in % DM and Na contents under salinity were significantly higher (P<0.05) in older compared to the younger segment. Ca content was lower in the salt-stressed cells than the non-stressed ones in both sampled locations (Tables 1 and 2) and Cl contents increased under salinity. These salinity-induced changes, demonstrate an age- related response to salinity of the growing cells, with elevation of salinity damages with increased age. It is therefore expected, that the transcriptome should be affected differently by NaCl at the two studied developmental stages, reflecting changes in the resistance or response to the stress. At each location, the tissue analyzed was homogenous, i.e., contained cells of similar developmental stage- this allowed identification of developmental related changes to the transcriptome.

**Table 1:** Effect of salinity on shoot biomass, leaf growth, and mineral contents in the leaf growing zone

**Parameter**	**Control**	**Salt**
Shoot fresh biomass (g)	6.68±0.37	3.80±0.16
Leaf elongation rate (mm h^-1^)	3.75±0.05	1.90±0.02

Effect of salinity on shoot biomass and leaf elongation rate (A), and on Relative elemental growth rate (REGR); % dry mater (DM), Na, Cl and Ca contents of growing leaf tissue from two locations along the growing zone: 15-30 mm and 30-50 mm from the leaf base (B). Plants were grown at 1mM NaCl (control) or 80 mM NaCl (Salt). Data are means ± SE (n=5).

**Table 2:** Effect of salinity on shoot biomass, leaf growth, and mineral contents in the leaf growing zone

	**Distance from leaf base (mm)**			
	**15–30 mm**		**30–50 mm**	
	**Control**	**Salt**	**Control**	**Salt**
REGR (h^-1^)	0.062±0.005	0.041±0.003	0.054±0.003	0.018.±0.001
DM (%)	7.35±0.19	10.20±0.40	6.13±0.20	9.11±0.29
Na (μmol g FW^-1^)	0.10±0.005	42.10±0.203	0.15±0.005	53.00±1.904
Cl (μmol g FW^-1^)	43.0±2.0	102.1±4.9	42.5±2.3	103.5±9.5
Ca (μmol g FW^-1^)	6.7±0.4	1.5±0.1	3.9±0.15.0	1.1±0.4

Effect of salinity on shoot biomass and leaf elongation rate (A), and on Relative elemental growth rate (REGR); % dry mater (DM), Na, Cl and Ca contents of growing leaf tissue from two locations along the growing zone: 15-30 mm and 30-50 mm from the leaf base (B). Plants were grown at 1mM NaCl (control) or 80 mM NaCl (Salt). Data are means ± SE (n=5).

### Identification of salinity-induced changes in gene expression in the growing cells of the maize leaf by cDNA subtraction library

To identify genes affected by salinity and possibly involved in the stress-induced growth restriction, PCR-based cDNA subtraction libraries were constructed from growing cells of two different developmental stages along the growing tissue of the maize leaf. The cDNA subtraction libraries were constructed for young cells from the center of the growing tissue, where the expansion rate is highest, and for older cells at a more distal portion of the growing zone, where the expansion rate is lower and the salinity effects are more pronounced. Forward and reverse subtractive hybridizations allowed identification of 72 differently salinity-affected genes in the growing leaf cells. Blastn and Blastp analysis based on nucleotide sequence or the corresponding amino acid sequence was performed in NCBI against *Zea mays*, rice or Arabidopsis. Identified sequences corresponding to genes are presented in Table 3.

**Table 3:** Functional classification, putative function (BLASTp (a) or BLASTn (b)) for the isolated genes

**GenBank Accession No.**	**GI**	**Protein ID**	**Annotation on*Zea Mays*Gene/Homolog**^**a**^	**Score**	**E Value**	**Region**	**FI ** **Salt/Control**
**Antioxidant Defense**							
EU968806	GI:195642911	http://ACG40924.1	Isovaleryl-CoA dehydrogenase mRNA	333	1e-90	15–30	2.96
BT018773	GI:54653554	http://NP_001150213.1	Acyl-[acyl-carrier-protein] desaturasea	401	3e-111	15–30	2.60
NM_001157202	GI:226492618	http://NP_001150674.1	Transaldolase 2	422	1e-116	15–30	2.81
EU958813	GI:195618201	http://ACG30931.1	3-isopropylmalate dehydratase small subunit 2	387	1e-106	15–30	3.17
NM_001155533	GI:226508813	http://NP_001149005.1	Aspartate aminotransferase	654	0.0	15–30	2.02
EU960286	GI:195621147	http://ACG32404.1	Acyl-CoA-binding protein	710	0.0	15–30	2.86
NM_001157741	GI:226499079	http://NP_001151213.1	Dihydroflavonol-4-reductase	545	2e-154	15–30	2.54
NM_001111889	GI:162459146	http://NP_001105359.1	Carbonic anhydrase	689	0.0	30–50	2.20
EU956253	GI:195613081	ACG28371.1	Phosphoserine phosphatase	765	0.0	30–50	0.46
**Structural**							
NM_001156318	GI:226500875	NP_001149790.1	Peptidyl-prolyl isomerase/cyclophylin	366	1-100	15–30	2.06
X68678.1	GI:829147	CAA48638.1	Cyclophylin	612	0.0	15–30	2.10
NM_001154900	GI:226497771	NP_001148372.1	Zn-finger, RanBP-type, cyclophilin-related protein	1090	0.0	15–30	2.13
EU977128.1	GI:195659556	ACG49246.1	UDP-glucuronic acid decarboxylase	1267	0.0	15–30	2.11
EU975955	GI:195657210	ACG48073.1	DNA-3-methyladenine glycosylase I	172	3e-42	15–30	3.14
NM_001174192	GI:293331322	NP_001167663.1	Tubulin alpha-3 chain	824	0.0	15–30	2.82
EU957585	GI:195615745	ACG29703	Retrotransposon protein	1186	0.0	15–30	2.09
NM_001153810	GI:226532905	NP_001147282.1	Ca2+-binding protein (EF-Hand superfamily)	669	0.0	15–30	2.04
NM_001155943	GI:226506681	NP_001149415.1	CTD-phosphatase-like protein	883	0.0	15–30	2.54
BT017876	GI:54652657		TIC21 iron ion transmembrane transporter	189	2e-47	15–30	2.85
NM_001111466	GI:162458261	NP_001104936.1	Dihydrolipoamide S-acetyltransferase	651	0.0	15–30	2.47
EU952983	GI:195606541	http://ACG25101.1	Threonine endopeptidase	278	4e-74	15–30	2.11
EU971467	GI:195648233	ACG43585.1	Calmodulin	577	8e-164	15–30	2.30
U29159	GI:902583	AAC49013.1	MubG1 ubiquitin gene	1227	0.0	15–30	3.39
EU963111	GI:195626797	ACG35229.1	Esterase precursor	852	0.0	15–30	2.29
NM_001174804	GI:293334320	NP_001168275.1	Translation initiation factor 4	357	7e-98	15–30	3.44
EU959748	GI:195620071	ACG31866.1	Elongation factor 1A	1426	0.0	15–30	2.82
EU968344.1	GI:195641987	ACG40462.1	60S ribosomal protein L3	370	9e-102	15–30	2.85
NM_001156254	GI:226502948	NP_001149726.1	60S ribosomal protein L5-1	800	0.0	15–30	2.30
EU970864	GI:195647027	ACG42982.1	40S ribosomal protein S19	682	0.0	15–30	2.96
EU958804	GI:195618183	ACG30922.1	40S ribosomal protein S4	436	1e-121	15–30	2.01
EU967930	GI:195641159	ACG40048.1	40S ribosomal protein S27a	710	0.0	15–30	2.07
EU952013.1	GI:195604601	ACG24131.1	30S ribosomal protein 3	401	3e-111	15–30	2.33
NM_001152240	GI:226505273	NP_001145712.1	SORBIDRAFTaSb=HSP70 cognate	933	0.0	15–30	2.08
BT085557	GI:238009749	ACR35910.1	Heat shock protein 70 cognate	837	0.0	15–30	2.19
NM_001154333	GI:226498819	NP_001147805.1	Heat shock 70 kDa protein 4	972	0.0	15–30	2.02
NM_001176042	GI:293336702	http://NP_001169513.1	TIDP2694, unknown function	451	4e-126	15–30	2.91
BT083594	GI:238005823	ACR33947.1	Transmembrane 9 superfamily protein 1 precursor	429	2e-119	15–30	2.01
EU948567	GI:195600921		Unknown	355	4E-97	30–50	10.78
NM_001157043	GI:226498795	http://NP_001150515.1	Dirigent-like protein pDIR9	344	7e-94	30–50	2.83
NM_001155737	GI:226504345	http://NP_001149209.1	1-aminocyclopropane-1-carboxylate oxidase ACC oxidase	442	3e-123	30–50	0.48
EU946392	GI:195598746	NP_001142128.1	Hydroxyproline-rich glycoprotein family protein^a^	662	0.0	30–50	2.01
BT064284	GI:223949794	ACN28981.1	Aspartic proteinase	429	2e-119	30–50	0.48
BT084696	GI:238008027	ACR35049.1	Elongation factor EF-Ts^a^	305	2e-82	30–50	0.47
BT061533	GI:223944292	ACN26230.1	Abhydrolase6, Hydrolase	446	2e-124	30–50	2.01
NM_001111648	GI:162461640	NP_001105118.1	Proline-rich protein; CL1298_1_ov	459	2e-128	30–50	0.25
NM_001147683	GI:226505411	http://NP_001141155.1	Oligopeptidase^a, Rc^	813	0.0	30–50	0.28
EU967200	GI:195639699	ACG39318.1	50S ribosomal protein L	263	1e-69	30–50	0.46
BT070196	GI:224036034	CN37093.1	18S ribosomal RNA gene	838	0.0	30–50	0.48
NM_001153810	GI:226532905	NP_001147282.1	Ca2+-binding protein (EF-Hand superfamily)	411	6e-114	30–50	0.48
EU957222	GI:195615019	ACG29340.1	Transposon protein CACTA	411	7e-114	30–50	0.24
NM_001138563	GI:212722439	NP_001132035	IAA15 - auxin-responsive Aux/IAA family member	571	4e-162	30–50	0.23
NM_001148300	GI:239050004	NP_001141772	PGR5-LIKE A^a^	412	2e-114	30–50	0.17
**Transcription Factors**							
BT063988	GI:223949202	http://ACN28685.1	drought-responsive factor-like transcription factor^a^	636	0.0	15–30	2.41
NM_001155696	GI:226532553	http://NP_001149168.1	RING finger, CHYzinc finger domain-containing	747	0.0	15–30	2.43
**Photosynthesis**							
EU967333	GI:195639965	http://ACG39451.1	Chlorophyll a-b binding protein CP24	241	5e-63	15–30	2.31
EU959735	GI:195620045	http://ACG31853.1	CP protein	239	2e-62	15–30	2.81
AY109815	GI:21213680		Magnesium chelatase subunit chlD^a^	619	1e-176	15–30	2.80
EU965631	GI:195636561	ACG37749	Ribulose bisphosphate carboxylase small chain	455	3e-127	15–30	2.19
BT069905	GI:224035452	http://ACN36802.1	AAA-metalloprotease FtsH^a^	920	0.0	30–50	0.46
EU965428	GI:195636155	http://ACG37546.1	Triose phosphate/phosphate translocator	1338	0.0	30–50	0.46
NM_001111878	GI:162463911	NP_001105348.1	Oxygen-evolving enhancer protein 3-1	520	1e-146	30–50	0.48
**Energy**							
EU953063	GI:195606701	http://ACG25181.1	Glyceraldehyde-3-phosphate dehydrogenase(GAPDH)	883	0.0	15–30	2.01
BT039975	GI:194701791	ACF84980.1	Cytosolic GAPDH	660	0.0	15–30	2.54
NM_001155853	GI:226507591	http://NP_001149325.1	ATP-citrate synthase	723	0.0	15–30	2.05
NM_001111964	GI:193211363	http://NP_001105434.1	Adenine nucleotide translocator (ANT2)	505	3e-142	15–30	2.60
EU96468	GI:195634658	http://ACG36798.1	Fructose-bisphosphate aldolase	161	2e-39	15–30	2.15
EU963078	GI:195626731	http://ACG35196.1	Vacuolar ATP synthase subunit G	167	6e-41	15–30	2.00
BT086232	GI:238011099	http://ACR36585.1	Vacuolar proton-inorganic pyrophosphatase	1042	0.0	15–30	2.01
NM_001155046	GI:226508897	http://NP_001148518.1	Malate dehydrogenase, glyoxysomal	1158	0.0	15–30	2.06
EU952363	GI:195605301	http://NP_001169698.1	2-oxoglutarate dehydrogenase E1	326	3e-88	15–30	2.23
EU955065	GI:195610705	http://ACG27183.1	Inorganic pyrophosphatase	1099	0.0	30–50	2.96
NM_001111961	GI:193211484	http://NP_001105431.1	Adenine nucleotide translocator (ANT1)	278	4e-78	30–50	0.48

GI-NCBI accession number, protein ID- protein NCBI accession number, region-the leaf zone from which the gene was isolated, FI-fold induction. Letters in uppercase above the gene name mark homolog to other species: a-*Arabidopsis thaliana*, Rc-*Ricinus communis*.

**Figure 2:**
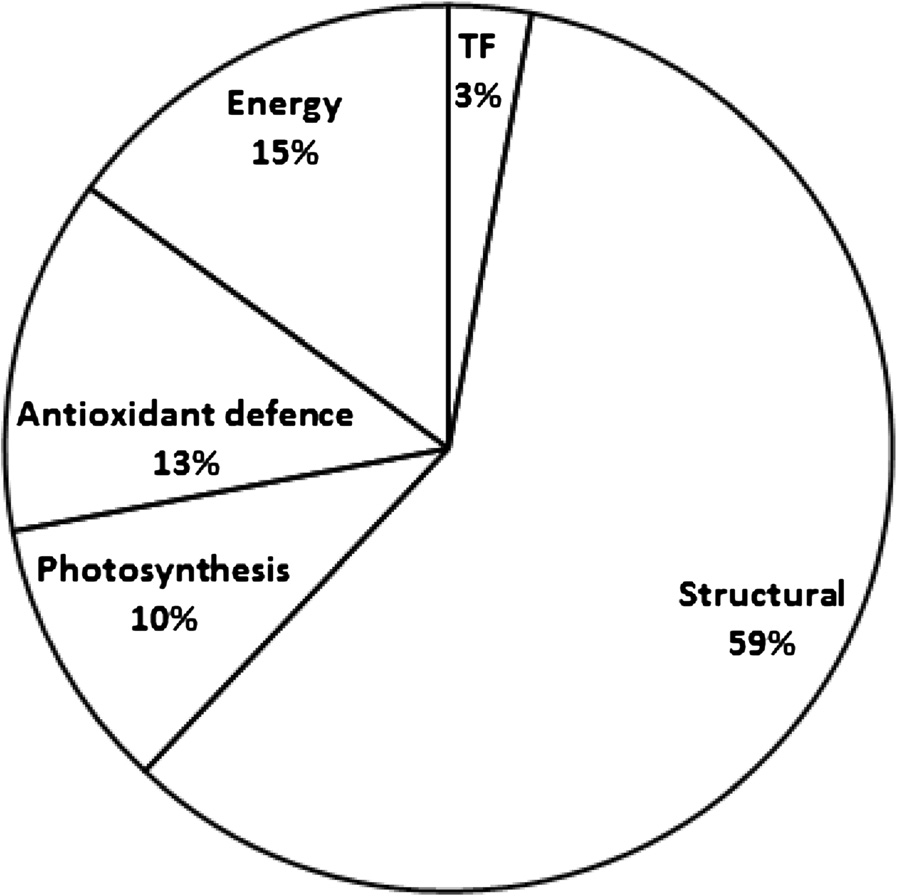
**Division of NaCl-affected transcripts obtained from the SSH library to functional groups.** The percentages in the pie-chart sections represent the ratio between various groups in the SSH library. The names of groups in the figure correspond to the group-names in Table 1. TF is transcription factor.

The identified genes represented transcripts involved in various biological pathways, and could be divided into several functionality groups: genes encoding to proteins involved in antioxidant defense (13%), electron transfer and energy (15%), structural proteins (59%), transcription factors (3%) and photosynthesis proteins (10%) (Figure 2; Table 3).

Genes encoding to structural proteins were represented by heat shock proteins (HSP) such as HSP70 that is known for its importance for stress tolerance, and ribosomal proteins that are known to be affected by salinity and play a structural role during the stress. Both these genes were induced in the region of maximal growth, i.e., the youngest segment sampled for the analyses, and were down-regulated in distal parts of the growth zone. The elongation factors EF1A and EF-Ts, which can both act as chaperones under the stress, were up-regulated in the cells location 15–30 mm from the leaf base, and down regulated at the distal 20 mm (30–50 mm from the leaf base). The structural gene hydroxyproline-rich glycoprotein, that can induce plant osmoprotection, was induced as well in the distal region of the growth zone (30–50 mm from the leaf base).

A number of genes that can affect various processes in the cell were affected. For example dirigent protein pDIR9 and 1-aminocyclopropane-1-carboxylate oxidase (ACC oxidase), that their proteins can affect rigidity of the cell-wall were up-regulated and down-regulated, respectively by salinity at the distal part of the growth zone. Energy metabolism was affected as well: most of the genes involved in energy metabolism were induced, and isolated from the library generated from the tissue located 15–30 mm from leaf base. For example, vacuolar inorganic pyrophosphatase that its hydrolysis supplies energy that drives H^+^ translocation into vacuoles, thereby aiding in generation of the transmembrane potential, was increased in the young tissue.

Genes involved in photosynthesis were affected differently in the two studied regions: at 15–30 mm from leaf base they were up-regulated by salinity, possibly representing the earlier development of the photosynthetic apparatus under salinity, while in the older cells from the location 30–50 mm from the leaf base they were down-regulated, probably representing NaCl damages to the fully photosynthetic tissue.

Intriguingly, a large share of the identified genes is associated with the plant antioxidant response and most of these genes were isolated from the 15–30 mm region, where growth is highest. For example, isovaleryl-CoA dehydrogenase (IVDH) that acts in the mitochondria, jasmonate-responsive (JR) genes such as 3-isopropylmalate dehydratase, and aspartate aminotransferase were up-regulated by NaCl in the young tissue segment. But over expression of antioxidant genes was also found in more distal region of the growing zone, for example for carbonic anhydrase. In addition to the genes listed in the antioxidant group to increase at the location 15–30 mm from the base, numerous other genes categorized in other groups (Table 3) can have an antioxidant activity as well. For example, the oxygen-evolving enhancer from the photosynthetic group, that involves in protection against photo-damages of the photosynthetic machinery increased in the location 15–30 mm, and so did the glyoxysomal malate dehydrogenase, that can stimulate glyoxysome activity and participate in energy generation. Glyoxysomes are a subclass of peroxisomes which play as well a role in antioxidant defense throughout fatty acid oxidation.

### Expression validation of differently affected genes

**Figure 3:**
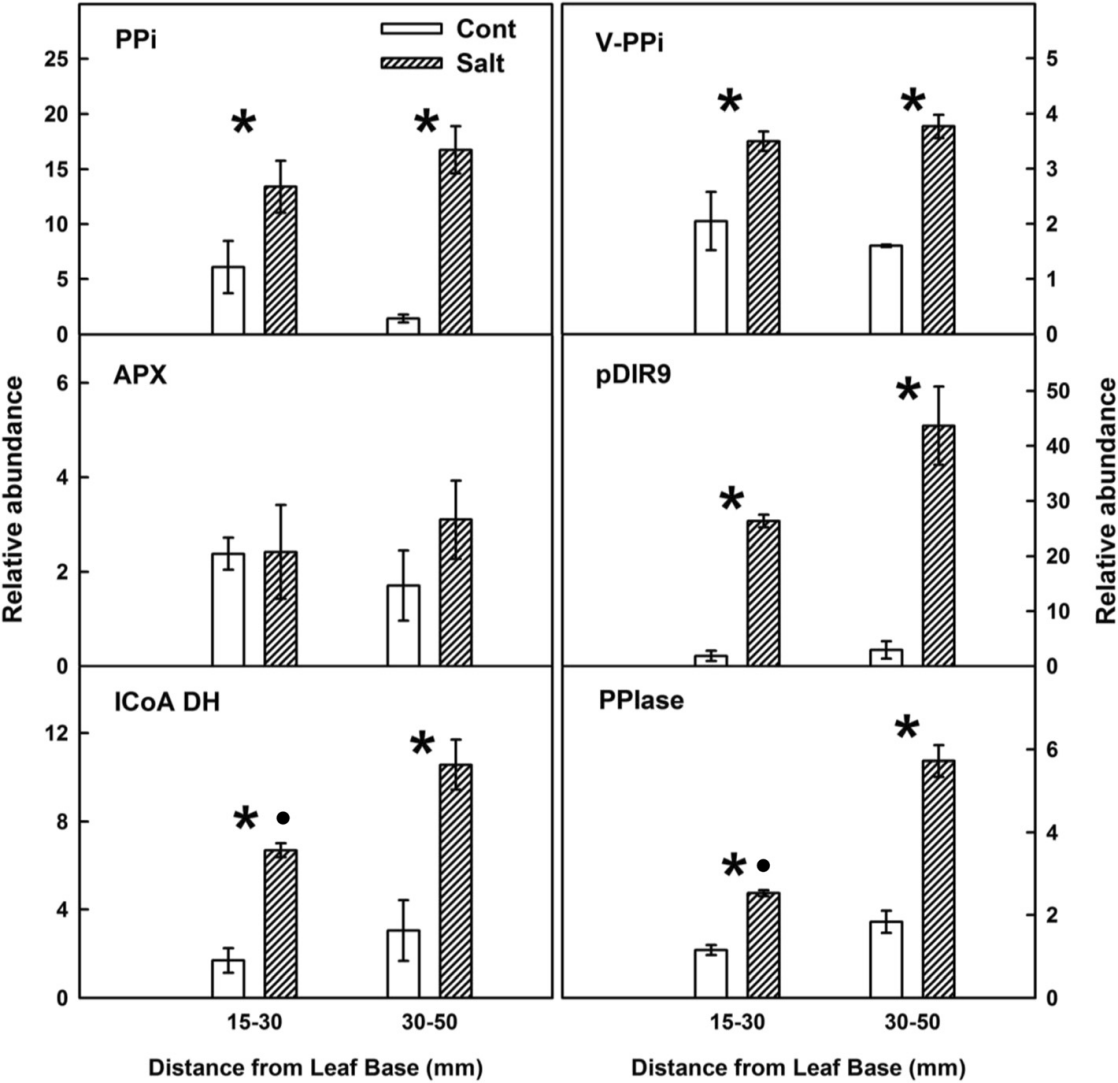
**Salinity-induced changes to selected transcripts in growing cells of the maize leaf.** Relative quantity determined by Real-Time PCR normalized to the amount of actin (Act-3, gi:21206665). White bars represent control conditions, and hatched box represent salt-stressed conditions at two locations in the elongation zone, 15–30 and 30-50 mm from the leaf base. Data are means ± SE (n=4-5). Asterisks represent significant difference between the salt stress and the control treatments for each equivalent position along the elongation zone (P<0.05). Filled dot above a treatment bar represent significant difference between young (15–30 mm) and more mature tissue (30–50 mm) (P<0.05) for this treatment. The names of the evaluated genes are specified in the figures: i*norganic pyrophosphate (PPi), vacuolar inorganic pyrophosphate (V-PPi), ascorbate peroxidase (APX), isovaleryl-CoA dehydrogenase (ICoA DH), peptidyl-prolyl isomerase (PPIase)*.

Real-Time PCR analysis was used for validation of the expression of several genes related to different groups as defined in Figure 3. The expressions of the genes encoded to inorganic pyrophosphotase (PPi), vacuolar inorganic pyrophosphotase (V-PPi), isovaleryl-CoA dehydrogenase (I-coA DH), dirigent-like protein (pDIR9) and peptidyl-prolyl isomerase (PPIase) was higher under salinity throughout the growing zone, in accord with the results obtained by the SSH. The expression level of ascorbate peroxidase (APX) (an antioxidant defense gene) that was not detected in the SSH library was found not to differ significantly between the control and salinity treatments in the Real-Time analysis as well. The expression level of ICoADH and PPIase under salinity was higher in the 15–30 mm segment compared to the 30-50 cm segment.

### Reactive oxygen species along the maize growth zone

**Figure 4:**
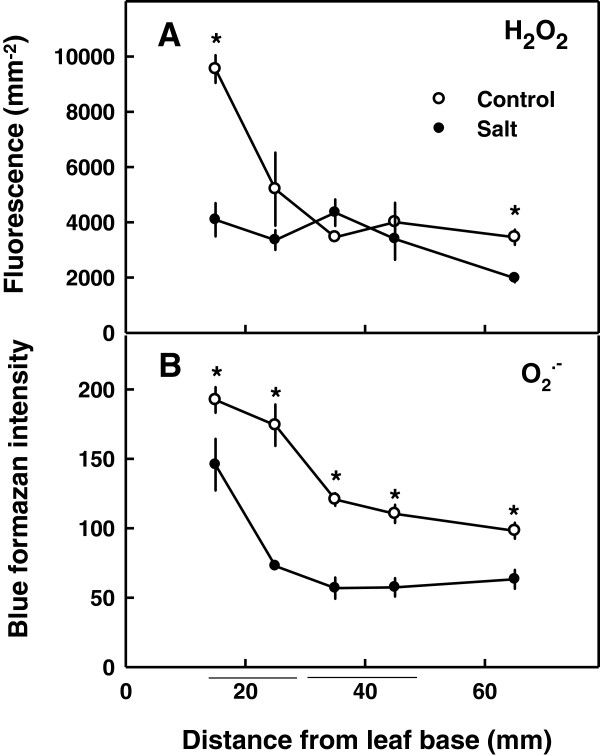
**Effect of salinity on concentrations of hydrogen peroxide (A) and superoxides (B) along the maize leaf elongation zone.** The plants were grown at 1 mM NaCl (control) or 80 mM NaCl (salt). Data are means ± SE (n=5). If not shown, error bars are smaller than the symbol size. Asterisks represent significant difference of salt stress treatment vs, control for each equivalent position along the elongation zone (P<0.05). The horizontal bars underneath the figure represent the two zones analyzed by SSH.

In light of the observed results, which identified 13% of the affected genes to involve in the antioxidative response, and the importance of reactive oxygen species (ROS) for cell growth, the oxidative state of the growing leaf tissue was studied. Spatial profiles of concentrations of two ROS throughout the developmental gradient that exists along the growing leaf tissue were analyzed (Figure 4). Hydrogen peroxide (H_2_O_2_) was higher at the leaf base, and at the older tissue at the distal part of the growth zone, in the control compared to the salt treatment (P<0.05). Throughout the remaining developmental gradient, the concentration was steady and similar for the two treatments. This is unlike, superoxides (O_2_^-^) that were lower throughout the growth zone under salinity. The largest difference between the control and the salt treatment was observed at the region where the cell undergoes the highest growth rate, 15–30 mm from the leaf base (Figure 4). Concentrations of both superoxides and hydrogen peroxide were significantly higher at the leaf base than at the end of growth zone in both the control and the salt treatments (P<0.05).

## Discussion

ROS was suggested to involve in cell elongation through an effect on cell-wall loosening [20-22], participation in signaling [23] and induction of degradation of macromolecules such as chlorophyll, membrane lipids, proteins and RNA under stress conditions or senescence [23,24]. Under salinity, reduction in ROS content was reported as one of the factors involved in leaf growth restriction [7,9], but ROS were also reported to increase under salinity [25] and to involve in salinity-induced leaf damages [16]. Consequently, ROS is considered today to have a dual effect under salinity, on one hand they are required for normal growth of young cells, but on the other they can induce damage especially in mature tissue. In the present study (Figure 4), in accord with previous studies [7,9], salinity was observed to reduce ROS levels in the growing cells. Various ROS scavengers are active in the plant cells, such as ascorbate peroxidase (APX), superoxide dismutases (SODs), glutathione S-transferase, and are known to involve in ROS detoxification during salt stress [2,7,23]. In the growing cells from the leaf base, APX and SOD activity was demonstrated to be significantly higher under NaCl [7]. The aim of the current study was to identify genes that are affected by salinity specifically at defined locations along the growing zone (i.e., correlate to defined stages of tissue expansion in the leaf) and may therefore involve in leaf growth restriction at defined stages of cell development. Due to the central roles suggested for ROS in facilitating cell growth and induction of cellular damage, it is not surprising that changes in ROS contents were identified in the growing cells, and that a considerable proportion (about 13%) of the affected genes belonged to the antioxidant group (Figure 2). Furthermore, the difference in sensitivity to salinity between cells from the two developmental stages evaluated, was reflected also in differences in antioxidant related proteins in the transcriptome.

Numerous antioxidant genes were found to be induced by salinity in the present study and most of these were isolated from the 15–30 mm region that was characterized by highest growth. At this region cell elongation is maximal (Figure 1), [4] and consequently there is a high demand for ROS for cell loosening [20]. The observed induction of genes involved in antioxidant defense at this early growth stage, resulted in lower ROS levels and consequently growth restriction observed previously by Neves-Piestun and Bernstein [4] and Rodriquez et al., [9]. For example, Isovaleryl-CoA dehydrogenase (IVDH), a key enzyme in the ETF/ETFQO complex in the mitochondria that induce an alternative respiration pathway that prevents ROS production in mitochondria under various conditions [26] was up-regulated by NaCl in the young tissue segment. ETF/ETFQO complex was reported to be induced under oxidative stress [27] and during dark induced senescence [28], and IVDH was shown to be essential for its activity [29]. Transaldolase, a key enzyme in the pentose phosphate pathway, which is the main NADPH-producing pathway. NADPH is involved in both the thioredoxin redox cycle and the glutathione redox cycle against ROS [30,31]. Additionally, expression of jasmonate-responsive (JR) genes was found to involve in resistance to oxidative stress [32]. 3-isopropylmalate dehydratase, which catalyzes the second step in the biosynthesis of leucine and is involved in the methionine chain elongation cycle for glucosinolate formation [33], was identified as a JR gene and involved in oxidative response [32]. The induced carbonic anhydrase was previously reported to be elevated during pathogen attack and consequently high ROS activity [34], and to act as an antioxidant and active player in plant disease resistance [35]. The induced oxidoreductase acyl-[acyl-carrier-protein] desaturase can have an antioxidant effect as well, because it can reduce O^-^ to two molecules of water [36]. The overexpression of this enzyme in tobacco under chilling stress significantly reduced ROS levels in the plant tissue and allowed elevated activity of antioxidant enzymes [36]. A phosphoserine phosphatase that is involved in amino acid production [37] can have oxidative effect as well since amino acids are known as antioxidants in plants [38] and phosphoserine phosphatase was reported previously to involve in stress tolerance [37]. Aspartate aminotransferase as well can be involved in antioxidant defense by the production of glutamate. It was previously reported that the glutamate metabolic flux was dominant in oxidative defense under water stress [39]. The acyl-CoA-binding protein was found to induce plant tolerance to various stresses including oxidative stress [40,41].

Additional genes can be involved in the antioxidant defense due to their activity in metabolic pathways. An overexpression of dihydroflavonol-4-reductase, which is involved in anticyonine production, led to cell-death resistance in rice due to reduction of hydrogen peroxide concentration [42]. Moreover the elevated NADPH levels observed in a rice mutant [42] can be supplied to the glutathione redox cycle for protection against ROS [43]. Surprisingly, important ROS scavengers such as superoxide dismutases (SODs), catalases (CATs), peroxidases and ascorbate peroxidase (APX), were not affected on the transcriptome level in the studied regions of the growth zone. Expression analysis for APX by Real-Time PCR supported this result (Figure 3). This is also in accord with a previous study from our lab which identified highest activity level of APX at the basal 15 mm region from the leaf base [7].

In light of the high expression of genes involved in antioxidant defense under salinity, it is suggested that they are the cause of the low ROS levels in the elongation region under salinity, and consequently the reduced cell-wall loosening. The plants have a very effective antioxidant system that facilitates maintenance of a tight ROS balance in the cell [2], and easily adapts to changes in ROS under abiotic stresses [2]. Indeed, in the distal part of the growth zone, only two genes involved in antioxidant activity were identified, and one of them was reduced under stress. This could result from the well documented reduction in photosynthetic activity under salinity [8]. Thereby, the plant adaptive strategy to prevent excess of ROS under stress condition by a massive expression of ROS scavengers, results in a negative effect on growth, i.e., growth restriction. From a plant survival, and thereby evolutionary perspective, growth restriction should be less damaging than the direct tissue damage which could occur by the stress-induced elevation of ROS.

In addition to genes belonging to the antioxidant defense system, various genes that can be classified into energy, photosynthetic and structural groups were affected as well by salinity. The elevation of these genes reflects the earlier maturation of the salt-stressed leaf tissues compared to the control; represent involvement in protection against the NaCl damages by an antioxidative defense; or participation in cell growth restriction mechanisms. For instance, reduced levels of ACC oxidase in salt-stressed plants in the 30–50 mm region that points to higher ethylene production [44], likely correspond to the difference in tissue age between the two treatments. I.e., the tissue found at the distal region of growth zone in salt-stressed plants is older than in the control treatment. This correlates with the observed elevation under salinity of the hydroxyproline-rich glycoprotein (HRGP), that is a component of the cell-wall produced in maize in an ethylene-dependent process, in the same region [45]. This elevation suggests an increase in cell-wall rigidity since HRGPs contain short rigid blocks of contiguous O-glycosylated hydroxyproline residues that involve in intermolecular cross-linking and cell-wall formation [46]. The dirigent protein pDIR9 was elevated as well under stress. Dirigent proteins are involved in lignin biosynthesis, act as physical barriers, have a strengthening role and repair damaged cell-walls [47]. Consequently they can increase rigidity under stress due to increased lignification and reduced cell-wall loosening. Effect of NaCl on these three proteins therefore points to earlier maturation of cells under salt stress and higher rigidity of the cell-wall under stress. The resulted higher rigidity under salinity will also reduce cell-wall loosening and hence the ability for cell enlargement. Elevation of these proteins under salinity in cells of the 30–50 mm region therefore correlates with the heightened growth sensitivity to salinity of this region compared to the younger region found 15–30 mm from the base (Tables 1 and 2). Earlier cessation of growth under salinity was demonstrated for the leaves studied in the present project (Figure 1) as well as in several other studies with monocot leaves [4,9,10].

Some of the genes identified by the SSH, such as the elongation factor EF1A or cyclophyllin, are known to have chaperones activity [48,49]. Salinity is known to promote protein dysfunction and reduce protein stability, and chaperones and heat-shock proteins are considered to involve in salt resistance by sustaining protein stability and function and prevention of protein aggregation [50]. In the young cells from the 15–30 mm region, three different Hsp70 were induced (Table 3), while in the 30–50 mm zone no genes that their products have chaperone functions were isolated and moreover, two isolated EFs were down-regulated pointing at lower protection against stress. Moreover, it was previously suggested that Hsp70 is involved in protein trafficking to peroxisomes [51] and consequently can affect peroxisome antioxidant activity. Again, these results correlate with the higher sensitivity to salinity of the older cells from the 30–50 mm region compared to the younger tissue from the 15–30 mm region. Overexpression of DnaK1 (a member of the Hsp70 group) in tobacco was demonstrated before to induce salt tolerance [52]. Taken together, these results demonstrate more protection against the stress in the 15–30 mm region compared to the 30–50 mm region, correlating to the difference in the extent of stress-induced growth reduction between these two regions.

Under salinity, increased energy demand for maintenance processes such as compartmentation and osmotic adjustment, coupled with reduced energy production via effects on the photosynthetic apparatus, might reduce energy availability in the plant. Sensitivity of growing cells to salinity might therefore be affected by localized energy-generating biochemical processes and genes involved in energy supply. As an adaptation mechanism, inorganic pyrophosphatase (H^+^-PPase) activity can be induced. H^+^PPase, can replace glycolytic ATP consuming enzyme reactions partially by reactions which utilize inorganic pyrophosphate (PPi) as an alternative energy source [53]. Induction of H^+^-PPase by NaCl increased with distance from the leaf base, i.e., cell age, along the growth zone. The smallest change was observed at the region of highest growth (15–30 mm from the leaf base). The higher expression of PPi at the distal part of the growth zone, where the cells are older, may suggest that the demand for alternative energy increases with prolonged exposure to the stress. At the same time vacuolar H^+^-PPase was isolated at the younger region. It can supply energy similar to PPi and can supplement energy demands at the region of highest growth. At 15–30 mm from leaf base (the center of the growing zone) additional genes involved in energy metabolism were induced by salinity, and overall this zone was affected by salinity more than the older zone, marking it as the primary affected cell developmental stage.

## Conclusions

The results observed in this study suggest that growth restriction under salt stress is induced by at least two processes. First, induced expression of genes encoded to products that acts as ROS scavengers results in reduction of ROS levels in the growing cells. This is supported by previous observations in monocot leaves [7,9]. The resulted reduction in ROS is involved in growth restriction under salt stress by reducing cell-wall loosening [9]. Second, induction of genes that enhance cell-wall rigidity reduces the capacity for cell expansion. Thereby, in the growth zone the cell-wall under stress may be more rigid and less attacked by ROS, resulting in cell growth restriction. Previously cell-wall rigidity was shown to increase by salinity in tips of maize roots [54] and under water deficit in growing leaves of maize [55]. Reduced sensitivity to salinity of younger cells from the center of the elongation zone, compared to older cells from more distal locations of the elongation zone was demonstrated for numerous grass leaves including maize (Figure 1) [7,17]. The differential transcriptomic results for the cells of the two developmental stages suggest that the higher growth sensitivity to salinity of the older cells might involve lower protein protection against the stress and higher cell-wall rigidity in the older cells. The detailed characterization of stress-inducible genes obtained in the current research increases our understanding of molecular mechanisms of salt stress effects in higher plants, and is also useful for directing programs geared at improving salinity tolerance- specifically towards optimization of the antioxidant response and cell-wall hardening processes in the growing cells of the leaf.

## Methods

### Plant material and growing conditions

Seeds of maize (Zea mays cv G.S. 46, Galilee Seeds, Haifa, Israel) were soaked in aerated solution (2 mM KCl and 1 mM CaCl_2_) for 6 h and than sown on moist vermiculite in plastic boxes. Plants were cultivated as previously described [4]. In short, the vermiculite was pretreated with 15 mM Ca(NO_3_)_2_ for 2 h, rinsed twice and later soaked in 0.1 concentration-modified Hoagland solution [19] for 2 h prior to sowing. The boxes were covered and kept in the dark at 25°C until d 4 when illumination started (400 μE s^-1^ m^-2^, 16-h photoperiod, relative humidity of 60% and 80% during the day and night, respectively). On d 7, plants with similar lengths of leaf 1 and 2 (80 ± 10 mm) were selected and transferred to aerated one-quarter-strength modified Hoagland solution [56]. Micronutrients were supplied as in one-half Hoagland concentration, except that iron was added as 50 μM Fe-EDTA and 20 μM Fe(NH_4_SO_4_)_2_ and Na level was elevated to 1 mM. Solution pH was adjusted to 5.7 with addition of KOH. Growth chamber conditions remained as described above. Salinization began with the transfer to hydroponics on day 7. At this time leaf 3 was not yet visible and leaf 4 was shorter than 10 mm. NaCl concentration in the growing medium was elevated in three daily steps (to 20, 50, and finally 80 mM) [4]. Control plants remained at a total concentration of sodium and chloride of 1 mM each.

### Plant growth analysis

#### Shoot and leaf development

Daily leaf length measurements were used for evaluation of shoot growth, and calculations of leaf elongation rates. Leaf length was measured daily with a ruler to the nearest 0.5 mm from the base of the plant to the tip of the leaf [57]. Leaf number 4, of 14- day old plants was selected for the experimental system in this study since the plastochron was lengthened under salinity from leaf 5 on and leaf 4 of the control and salt-stressed plants emerge above the whorl of encircling older leaf sheaths on the same day. Selection of this leaf therefore prevents complications arising from interpretation of experimental results from leaves which differ in developmental stage [4]. Furthermore, on this day, this leaf was at the rapid phase of elongation and thereby contained cells at all stages of development: from dividing cells in the basal meristem located near the point of leaf attachment to the node, to growing and mature cells at more distal locations.

For all measurements conducted in the study the plant tissue was sampled for analyses on day 7 after the beginning of salinization, when the plants were 14-days-old.

### Biomass determination

For biomass determination, growing leaf segments located at the region of highest growth (15–30 mm from the leaf base) and the distal part of growth zone (located 30–50 mm from leaf base), which are equivalent to the locations used for the SSH analyses were excised from the leaf. The age of cells in the center of the 15–30 mm segment from the control and salt treated plants was 30.6 h and 48.6 h, respectively, and 39.6 h and 61.2 h respectively in the 30–50 mm segment (Neves-Piestun and Bernstein, unpublished). Four replicated leaves from different plants were aligned and cut together, so that 4 cut segments were combined into samples by position. Fresh biomass was recorded immediately following excision from the plant. Dry biomass was measured after drying at 60°C for 48 h and cooling for 24 h in a desiccation chamber. Weights were measured with a Precisa 40SM-200A balance (Zurich, Switzerland) to the nearest 10 μg. The fresh and dry weights were used for calculations of % dry weight, %DW, of the tissue.

### Mineral analysis of the plant tissue

Tissue located 15–30 mm and 30–50 mm from the leaf base of leaf 4 was sampled for mineral analyses as well. Contents of Ca, as well as the salinity sources (Na, Cl) in the leaf tissue were determined as previously described [19]. Segments from 5 replicated leaves from different plants were combined by position for each sample. Fresh and dry weights were recorded with a Precisa 40SM-200A balance (Zurich, Switzerland) to the nearest 0.00001 g, and percentage of water in the tissue was calculated. In short, for the analysis of Ca, the dried plant samples were digested with HNO_3_ and HClO_4_ (65% and 60%, respectively). The extract was analyzed for Ca, by inductively coupled plasma atomic emission spectrometry (ICP-AES), (Spectro, Kleve, Germany). For the analysis of Na and Cl the dry tissue was extracted with a dilute acid solution containing 0.64% HNO_3_ and 10% CH_3_COOH. Samples were analyzed for chloride by potentiometric tritation (Buchler chloridmeter, New Jersey, USA) and for sodium by flame photometry (Instrumentation Laboratory, USA).

### RNA extraction, cDNA production, PCR, cloning of PCR products, subtraction library

For the subtraction libraries, the region of highest growth (15–30 mm from leaf base) and the distal part of growth zone (located 30–50 mm from leaf base) were selected. For quantitative Real-Time PCR analyses, the growing zone was sectioned into 4 regions: 20–25, 35–40, 50–55, 60–65 mm from leaf base. RNA was extracted by Tri-reagent (Sigma-Aldrich Co) according to the manufacturer’s instructions. For removal of genomic DNA from RNA preparations DNAse (Fermentas Inc, Maryland, USA) treatment was produced accordingly to the manufacturer’s instructions. cDNA was produced by verso cDNA kit (Thermo Fisher Scientific Inc., ABgene House, Surrey, UK) accordingly to the manufacturer’s instructions. A PCR-select cDNA subtraction kit (Clontech Laboratories Inc., Mountain View, USA) was used for the generation of the subtraction library, and screening of the subtraction library was performed with a PCR-select differential screening kit (Clontech Laboratories Inc., Mountain View, USA). The forward subtraction used tester cDNA obtained from mRNA of salt-treated tissues and driver cDNA from control treatment. In the reverse subtraction, the tester cDNA was obtained from mRNA of control treatment and driver cDNA from salt-treated tissues. Dot-blot analysis was performed with a PCR-select differential screening kit (Clontech Laboratories Inc., Mountain View, USA). Around 90 clones from these libraries were sequenced and gene identities were determined by sequence comparison to the nonredundant GenBank database using BLASTn, using default parameters. In instances where an unannotated match was obtained, BLASTp (http://www.ncbi.nlm.nih.gov) searchers were conducted and sequence homology information was used to assign putative identities.

### Quantative real-time -PCR

Real-Time PCR was performed with Absolute Blue QPCR Sybr Green ROX mix (Thermo Fisher Scientific Inc., ABgene House, Surrey, UK) at Mx3000P® QPCR System (Stratagene, La Jolla, CA, USA). All reactions were performed with 4–5 independent biological repeats, with triplicate testing for each replicate. The relative abundance of transcripts was normalized with actin. Data were analyzed using the MxPro-Mx3000P v.4 and relative quantity (RQ) was calculated from Real-Time PCR data by 2-∆∆CT. Primer are listed in Table 4. Statistical analysis was performed in JMP 5.0.1 (SAS Institute Inc, Raleigh, NC, USA).

**Table 4:** List of primers used for the Real-Time PCR analysis

**Gene name**	**GI**	**Forward primer 5**^**′**^**-3**^**′**^	**Reverse primer 5**^**′**^**-3**^**′**^
*Actin*	99030435	TGCTGAGCGAGAAATTGTCAGGG	TTCCATGCCAATGAAGGATGGCT
*APX*	600115	ATCGCCGAGAAGAATTGCG	GGTTCTTCATGGTGCCGAA
*PPi*	195610705	GAGCTCTCGTTGGCCTGATTT	ACGAAGAAGCATGGTCACAGC
*V-PPi*	238011099	GTGTTGCAATTGGTCTGTGG	CTGCAAGAATCTGCAACGTC
*pDIR9*	226498795	CCACTTCTTCTTCCACGACAC	TCCATCACGTTCACCATCC
*PPIase*	226500875	AGCTTTGCACCAAGGTTCTG	TCAGGATCGATTTCCAGTGC
*IcoA DH*	195642911	ATGTCGTCAGCATGAAGTGC	CGTAAACAACCAGTGTCTGAGC

*Inorganic pyrophosphate (PPi), vacuolar inorganic pyrophosphate (V-PPi), ascorbate peroxidase (APX), isovaleryl-CoA dehydrogenase (ICoA DH), peptidyl-prolyl isomerase (PPIase)*.

### Determination of content of reactive oxygen species

#### Hydrogen peroxide (H_2_O_2_) determination

H_2_O_2_ level in the tissue was analyzed along the developmental gradients of leaf 4 at day 14. Leaves were attached to a glass plate and tissue disks, 6 mm in diameter, were sampled from several regions: 10–20 mm, 20–30 mm, 30–40, 40–50, 60–70 mm from leaf base. The disks were washed with double distilled water and immediately analyzed for H_2_O_2_. The hydrogen peroxide levels were determined using 2,7- dihydrodichlorofluorescein diacetate (H_2_DCF-DA) [58]. Solution of 25 mM was prepared in methanol and kept at −20°C pending use. Discs were transferred to small wells of ELISA plates containing 200 μl of fresh MES buffer (50 mM; pH 6.2) and 10 μM of H_2_DCF-DA. Following incubation for 30 min at room temperature, fluorescence was measured with a microplate fluorescence reader FL600 (Bio-Tek, Vermont, USA), using 485 Ex and 530 Em filters [58]. Results expressed in fluorescent units and each data point represent an average ± SE (n=5).

#### Superoxide determination

A 70 mm segment was sectioned from the base of leaf 4 of 14-day-old plants for superoxide determination. Immediately following excision from the plant, the tissue was gently washed for 30 sec in DDW and subjected to staining for superoxides. Superoxides accumulation in tissue was determined with nitrotetrazolium blue (NBT, Sigma-Aldrich Co, St. Louis, USA) [16], which reacts with O_2_^·-^, producing a blue formazan precipitate. The segment was gently vacuum infiltrated (2 min) with 0.01% NBT solution and incubated in the dark in the same solution for 2 h at 30°C under very slow shaking. To determine that this staining was attributed to the formation of O_2_^·-^, MnCl_2_ (10 mM), a highly effective O_2_^·-^ dismutating catalyst agent [16], was added together with NBT as a control. After staining, the chlorophyll was removed from the tissue by boiling the segments in 9:1 solution of ethanol and glycerin for 10 min. Color density was checked with ImageJ 1.42q (NIH, USA, http://rsbweb.nih.gov/ij/). Results are expressed as color density and each data point represent an average ± SE (n=4).

### Statistical analysis

Results are expressed as means ± standard errors (SE). Statistical analysis was performed using JMP 5 software (SAS Institute Inc., 2002, Cary, NC, USA). Data were subjected to one-way ANOVA analysis and Tukey honestly significant difference for comparison of means.

## Competing interests

The authors declare that they have no competing interests.

## Authors’ contributions

MK performed the SSH analysis, RT PCR analysis, ROS analysis and drafted the manuscript. NB conceived of the study, performed the growth kinematics analysis, mineral ion analyses and revised the text. Both authors read and approved the final manuscript.
